# Unwanted loneliness, food practices, and Mediterranean diet adherence among older adults in Barcelona city (Spain)

**DOI:** 10.3389/fnut.2026.1947655

**Published:** 2026-10-08

**Authors:** F. Xavier Medina, Laura Esquius, Alicia Aguilar-Martínez

**Affiliations:** FoodLab Research Group, Faculty of Health Sciences, Open University of Catalonia (UOC), Barcelona, Spain

**Keywords:** aged, feeding behavior, home environment, loneliness, Mediterranean diet, qualitative research

## Abstract

Progressive population aging and the rise of single-person households have profoundly transformed the dynamics of daily commensality. This article presents a phenomenological mixed-methods study exploring the food practices, habits, and emotional meanings of older adults (aged 65 and over) living alone. Through in-depth interviews with 12 participants, combined with an assessment of their adherence to the Mediterranean Diet and a visual analysis of their home food environment, a restructuring of culinary and dietary routines is evidenced. The results reveal a functional simplification aimed at avoiding the effort of cooking for oneself, the abandonment of “social foods” due to a lack of companionship, and a moderate-to-low dietary adherence (6.75/14). The analysis indicates that even when the home food environment is health-promoting, the lack of commensality and culinary apathy disrupt dietary habits. In conclusion, eating in solitude threatens the sustainability of the Mediterranean diet pattern, highlighting the need for public health interventions focused on mitigating social isolation.

## 1 Introduction

The act of eating transcends mere biological necessity to constitute a deeply social, cultural, and emotional phenomenon (1). According to the Merriam-Webster Dictionary, the word commensality means “the practice of eating together” or “a social group that eats together.” This practice of sharing food appears to be universal. It is present across all cultures, locations, and historical periods. In a broad sense, as French sociologist Michel Maffesoli notes, we rarely eat alone. In this regard, eating together has always been one of the foundations of any community (2, 3). In the Mediterranean context, eating represents the core of socialization and community cohesion (commensality, conviviality) (4, 5). This intangible cultural pillar was recognized by UNESCO when it declared the Mediterranean Diet as an Intangible Cultural Heritage of Humanity in 2010 (6, 7).

The aging of contemporary societies is already a commonplace in the literature (8). In a country like Spain, the Aging Index has risen from 37.35 to 120.46 in the last 30 years (1978–2018), with the percentage of older people in the total population reaching 19.1%. Other countries in the Mediterranean basin show very similar and even slightly higher figures. This is the case with Italy (22.6%), Greece (21.8%), or Portugal (21.5%) (8). The importance of this data is not based solely on a trend that appears to be increasingly established over time, but on the challenges it implies for public health. Of particular importance are those derived directly from loneliness and social isolation. Beyond the numbers, these challenges prove difficult to address from a social, cultural, or psychological point of view (9). In a recent national survey, conducted in 2024, a significant portion of the Spanish population reported experiencing loneliness. This includes 20% of all adults, 14.5% of those aged 65–74 years, and 20% of those aged 75 years and over (10).

Living alone in old age entails multidimensional challenges that significantly alter dietary behavior. Several studies have documented that older adults living without company tend to skip meals and reduce their variety of fresh products. They also experience a general loss of enjoyment in food, adopting an approach that is often summarized by the expression “eating to not die” (11). Although the literature has explored the relationship between loneliness and the risk of malnutrition (12), it is necessary to expand the evidence regarding the different personal, community, and social factors that influence social isolation and loneliness. Furthermore, more evidence is required on the relationship between loneliness, dietary habits, and the food environment (13–15).

The Home Food Environment (HFE) acts as an invisible barrier or facilitator in daily decision-making (16, 17). Visual stimuli in the home determine dietary behavior through unconscious nudging mechanisms (18). Typical examples include the accessibility of a fruit bowl or, conversely, the exposure of ultra-processed products on countertops.

The aim of this study is to explore in depth how loneliness impacts the food choices, shopping routines, and culinary practices of older adults over 65 years old living alone in Barcelona City. While this research is part of a broader comparative national project, Barcelona was strategically selected as a metropolitan case study due to its critical demographic profile. According to official municipal and regional statistical reports (19–21), adults aged 65 and over constitute 26.2% of the city's total population, presenting a severe aging index of 180. Furthermore, over 18% of individuals in this age group—rising to 30% among those over 80—live alone, making it an ideal urban environment to investigate the intersection of metropolitan isolation and dietary practices. Additionally, this study aims to contrast the subjective perception of these individuals regarding their own diet with objective measures of Mediterranean Diet adherence and the quality of their physical home food environment.

## 2 Materials and methods types

### 2.1 Study design and sampling

A cross-sectional study with a convergent mixed-methods design and a community-based approach was conducted. The mixed ethnographic research (22) prioritized the qualitative-phenomenological depth of the participants' lived experiences (23–25). The methods combined in-depth semi-structured open interviews, participant observation (26) and direct observation. This perspective was supported and triangulated by validated questionnaires on their dietary patterns, and the physical objectification of the domestic space through photodocumentation related to food and domestic spaces of storage, transformation and consumption.

Fieldwork was conducted across different neighborhoods in the city of Barcelona. This research is part of a broader project developed across multiple regions of Spain. This larger project was designed to compare results between areas with different socioeconomic characteristics and varying levels of urbanity-rurality. Recruitment was performed using purposive non-probability sampling. The sample comprised 12 older adults over 65 y/o (7 women, 5 men; age range: 66–87 years) who lived alone in their homes within the municipality of Barcelona. The participants' trajectory of loneliness varied from recent widowhood (7 months) to divorce or several decades of singlehood. The sample limit was established in response to indications of information saturation (22, 23).

Inclusion criteria required participants to be 65 years or older, live alone in their usual residence in Barcelona, and autonomously manage food shopping and preparation. Exclusion criteria included a diagnosis of dementia or pre-dementia, or a daily reliance on social services for meals that would limit their capacity to decide on their own diet.

The study was conducted in accordance with the Declaration of Helsinki and the ethical principles for research involving human subjects and the processing of personal data. The project received approval from the Bioethics and Biosafety Committee of the University of Extremadura (176/2024). All participants signed an informed consent form for the recording and storage of interview audio and for taking images of their private kitchen environment. Participant confidentiality was guaranteed throughout the process by using alphanumeric codes for all data analysis.

### 2.2 Data collection procedure

Data collection was carried out between March and September 2025. In-depth open semistructured interviews took place during face-to-face sessions at the participant's home, conducted by a researcher trained in qualitative methodologies and in-depth interview techniques. First, the Mediterranean Diet Adherence Screener (MEDAS), validated for the Spanish population, was administered verbally in an assisted format (27). Scores range from 0 to 14 points, classifying adherence as low ( ≤ 5), moderate (6–8), or high (≥ 9). After establishing rapport, in-depth interviews were conducted using a previously piloted guide. The interview guide explored key dimensions: (1) food shopping practices and locations; (2) culinary preparation strategies; (3) intake habits and schedules; (4) symbolic and emotional meanings, subjective narrative memories of their past culinary routines (e.g., when they used to cook for a family); and (5) the impact of social life, companionship, and loneliness in the home. Interviews had an average duration of 55 min, were audio-recorded, and were transcribed verbatim. The interviews were conducted in Spanish or Catalan, depending on the interviewee's native language or preferred language. The English translations in this article were done by the authors, who attempted to preserve the original contexts and linguistic nuances whenever possible.

To evaluate the current physical context, photographs were taken at the end of the interview and also during the observations or participant observations. Among others, three standardized digital images were obtained per household to capture the present-day availability of foods and kitchen infrastructure: (a) a frontal view of the open refrigerator (including shelves and the lower crisper drawer), (b) a frontal view of the open pantry or food storage cabinet, and (c) a general view of the kitchen. To transform the images into usable quantitative data aligned with the Mediterranean pattern, the HFE-MED-PHOTO instrument was designed and implemented. This tool was inspired by validated home food environment inventories (17). Two researchers independently evaluated the obtained images. Minor discrepancies were resolved by consensus through the involvement of a third researcher. The variables included in the visual audit cover the storage of vegetables and fruits; the presence of dairy and protein foods, including nuts and legumes; the existence of olive oil; and logistical and sustainability factors (such as the presence of homemade lunchboxes, ready-to-eat foods, or a microwave). The scoring range for this instrument was −7 to 16 points. Three classification thresholds were established: Risk Environment ( ≤ +1 point), Vulnerable Environment (+2 to +8 points), and Promoter/Sustainable Environment (≥ +9 points). The detailed breakdown of scores, variables, and the coding matrix of the tool is available in Supplementary File 1.

### 2.3 Data analysis

Interview analysis was performed using thematic analysis (28) with NVivo 14 software. To integrate the data (29), qualitative interviews were linked to each participant's attributes (age, neighborhood, MEDAS, and HFE-MED-PHOTO). This linkage allowed for a systematic cross-referencing of the qualitative narrative with the rest of the quantitative variables.

## 3 Results

The results of this study reveal distinct behavioral patterns regarding how involuntary loneliness reshapes food practices in older adults. Five key themes emerged from the interviews: face-to-face dynamics in food acquisition; pragmatic simplification in culinary preparation; the flexibilization of dietary patterns; the social, emotional and symbolic dimension of food; and the relationship of diet and health. Table 1 shows a summary of the main results.

**Table 1:** Thematic categories of the qualitative analysis, subcategories, and illustrative quotes (Verbatim).

Main category	Subcategories	Illustrative quotes
1. Food acquisition (Shopping)	1.1. Face-to-face shopping and rejection of the digital environment	“If there are things that are going to be heavy for me, I take my shopping cart and I go with my shopping cart. I am very analog, very analog, and as I tell you, very—underline the word very—very analog.” (E01)
	1.2. Economic constraints and the pension/price sensitivity	“Salmon, I like it a lot... I buy it occasionally because of the price. As I told you, I am retired, unfortunately... my day-to-day life, economically, is a little rigid.” (E10) “It was almost 7 euros a kilo, 6-something... it's not so much that you can't afford it, but rather that you just don't feel like buying what you want.” (E04)
2. Culinary preparation (Cooking)	2.1. Simplification and apathy	“My cooking is super fast. I mean, I don't sit down to cook. I make it quick... I have my fantastic Thermomix that makes me purees that are to die for.” (E11)
	2.2. Food preservation and ready-to-eat meals	“I don't cook every day. I mean, when I cook, I cook a big pot, and then I make tupperware containers. Because if I had to cook for one, I would eat a salad and a steak every day.” (E08) “Look, cannelloni, I'm not going to start making cannelloni for myself. I have to buy a tray [...] You put it in the oven and that's it.” (E10)
	2.3 Gender differences	“When my husband was alive, meals were more substantial [...] Now I make a little rice for myself [...] it gives me a sense of freedom, of not having to do anything.” (E02) “Since I don't have to give explanations to anyone, I don't have to ask, ‘Do you feel like making... I don't know, whatever?' and that's it.” (E12)
3. Dietary patterns (Eating)	3.1. Schedule flexibility	“I have been not having dinner for 15 days now, and it's going well for me [...] (E11)
	3.2. Television as a background companion during meals”	“It is true that I put the TV on in the background for company, or sometimes I put on very soft music, I put it on for company, usually the television more.” (E10) “I run to the dining room in front of the TV, and I start drinking water. I get hungry! And I cannot eat, so I force myself.” (E11)
4. Social aspects and emotional and symbolic dimension	4.1. Restaurants as a strictly social act	“Eating out alone sometime? Never, never. I mean, here I nourish myself, I eat, but if I go out to eat it's to, it's to socialize.” (E12)
	4.2. “Social foods” and loss of meaning	“I don't eat cockles alone... or buy them, even if I like them. I know it's silly, but there are things to eat that are social.” (E06)
	4.3. Celebrations	“Apart from that, it's just that Christmas is no longer the same for me, you know? Rather, I am wishing for it to be over [...] that's when you are most with your family and you have a lot of memories.” (E05)
5. Health and food	5.1. Diet as self-care	“I can eat a plate of vegetables, I mean, I force myself to eat it as if it were medicine.” (E08) “I look at the labels a lot. [...] The sugar, the salt... Yes, I look at all of this quite a bit.” (E05)
	5.2. Importance of pleasure	“I am not interested in life at all in general, okay? I mean, if I have to die three years earlier because I ate a steak, I don't care.” (E03) “My sweet portion, which I will absolutely never give up, is dark chocolate [...] it's like saying: I've finished eating.” (E12)

### 3.1 Food acquisition dynamics and economic constraints (Shopping)

Food shopping becomes a frequent, almost daily routine. Participants use physical shopping as an intentional strategy to establish a daily structure, leave the house, and engage in social interactions. The quantities purchased are small, for quick consumption, and are purchased frequently. Online food shopping and home delivery services are unanimously rejected. This rejection is motivated by the desire to preserve physical mobility and personally evaluate fresh produce. Material conditions, specifically pensions, create differences among participants. Those with low pensions experience restricted access to fresh products, especially fish, and typically purchase based on discounts or rely heavily on supermarkets. However, for others, shopping for a single person allows them not to deprive themselves of certain treats, although the high cost of some foods occasionally causes them to avoid these items out of frustration rather than a lack of liquidity.

### 3.2 Culinary preparation (Cooking)

A generalized process of pragmatic simplification is observed in culinary preparation. Cooking for oneself generates a lack of motivation (apathy), which discourages traditional preparations that require significant time or multiple cooking utensils. Furthermore, the experience of this culinary solitude is influenced by gender: for many women, who historically cooked to accommodate their husbands' tastes, living alone represents a liberation from the burden of caregiving, translating into a lack of interest in slow, complex stews. Conversely, for men, it represents a late-life learning process, often centered on quick and repetitive survival cooking. In all cases, there is a reliance on ready-to-eat meals or cooking larger quantities for several days (*batch cooking*). This approach also helps to avoid food waste, even if it forces participants to consume the same dish for consecutive days. Paradoxically, the fear of fresh food spoiling (especially fruit) acts as a barrier, discouraging its purchase and indirectly reducing frequently its availability in the home.

### 3.3 Spatial and temporal destructuring (Eating)

Solitude at home grants absolute temporal autonomy, which frequently leads to the normative destructuring of mealtimes. The traditional structure of a first course, second course, and dessert has been lost, with a predominant shift toward a single-plate meal consumed quickly (10–15 min). The adoption of meal-skipping practices stands out, most commonly skipping dinners or substituting them with minimal intake (such as an herbal tea or a yogurt). Spatially, the act of eating is rigidly ritualized: participants always eat in the exact same place (the kitchen table or a specific corner of the dining room), routinely using their same everyday tableware. The silence within the home is perceived as an unpleasant barrier that is mitigated by eating with the television or radio turned on to provide company.

### 3.4 Food and health

The relationship with food reveals a constant tension between diet understood as a self-care tool for health and the defense of individual pleasure (10). On the one hand, healthy eating is sometimes assumed as an obligation, which compels individuals to force the intake of vegetables or to strictly monitor labels (e.g., the amount of salt and sugar). On the other hand, the hedonic component of food is strongly defended; immediate gratification and individual taste are prioritized over longevity or strict medical guidelines, while also preserving small, non-negotiable daily rituals.

### 3.5 Mediterranean diet adherence (MEDAS) and the home food environment (HFE-MED)

Assessment using the MEDAS questionnaire indicates that the sample exhibits a moderate-to-low level of adherence, with a mean score of 6.75 out of 14 points. All participants reported using olive oil as their primary cooking fat, and 91.7% prioritized the consumption of white meat over red meat. However, a severe deficit was observed in vegetables (only 2 out of 12 reached the recommended two daily servings) and fresh fruit (only 25% consumed three or more pieces per day). The evaluation of photographs of kitchens, refrigerators, and pantries using the Home Food Environment index (HFE-MED) yielded a group mean score of 5.83 out of 16 points. Only 3 out of the 12 participants (E01, E02, E05, E09) achieved an HFE-MED score equal to or greater than 9, which is considered a promoting environment for the Mediterranean Diet. Among these trhee participants, only one individual (E02) achieved high adherence to the Mediterranean Diet (with a MEDAS score of 9).

### 3.6 Quantitative integration and triangulation

To objectively validate these narratives, the qualitative findings were integrated with the quantitative metrics from the Mediterranean Diet Adherence Screener (MEDAS) questionnaire and the HFE-MED-PHOTO home visual audit matrix.

The qualitative triangulation delineated three distinct profiles encompassing all 12 participants: (a) High Adherence / Promoting Environment Profile represented exclusively by E02 (HFE-med 12, MEDAS 9), the only informant who cooks daily for others (her granddaughters). In this case, caregiving and companionship act as a protective shield for the diet, promoting the actual consumption of the food available within the household; (b) Moderate Adherence / Mixed Environments Profile: Comprising E01, E03, E05, E06, E07, E09, and E10. This group maintains moderate dietary adherence through different pathways. While some participants (e.g., E01, E05, E09) display a strong desire to eat healthily and maintain good home food environments (HFE-med 10 and 11), yet whose diets score lower due to pragmatic adaptations dictated by solitude (for example, avoiding perishable fruits due to the fear of them spoiling),others face structural or financial constraints in their storage spaces (e.g., E03, E06, E07, E10) but retain sufficient culinary inertia or informal family support to prevent a total dietary drop. (c) Low Adherence/Vulnerable-Risk Environment: Comprising E04, E08, E11, and E12. These participants present lowest dietary adherence and severely compromised home food environments (HFE-med 5 or lower, reaching negative risk scores in E08 and E12). This group is characterized by extreme culinary simplification, a refusal to use the stove, a high reliance on ready-to-eat meals, and a view of eating as a mere mechanical chore.

The integrated triangulation matrix (Table 2) crosses these metrics with the dominant qualitative trajectories, revealing gender-based differences.

**Table 2:** Triangulation matrix.

Participant	Gender	Age district	MEDAS Score	HFE- PHOTO score (Profile)	Dominant qualitative trajectory	Concordance/Discordance synthesis
**E01**	Female	74/Gràcia	6 (Moderate)	10 (Promotor)	Resilient neighborhood shopping but extreme daily cooking apathy.	**Discordant:** High structural food availability via local shops, neutralized by severe cooking avoidance.
**E02**	Female	72/Eixample	9 (High)	12 (Promotor)	Sustained caregiving role; cooks traditional fresh meals for granddaughters.	**Concordant:** The presence of a social recipient protects both kitchen infrastructure and dietary intake.
**E03**	Male	85/Sants	7 (Moderate)	6 (Vulnerable)	Late-life singlehood; high reliance on non-perishable canned items and daily beer.	**Concordant:** Functional adaptation with structural deficits in fresh fruits and vegetables.
**E04**	Male	76/Horta	5 (Low)	2 (Vulnerable)	Active memory blocking; absolute exclusion of wine due to solitary stigma.	**Concordant:** Psychological distress restricts the integration of traditional social dietary markers.
**E05**	Female	68/Ciutat Vella	6 (Moderate)	11 (Promotor)	Active retirement; uses physical shopping paths as urban social therapy.	**Discordant:** Excellent physical food environment countered by single-plate simplification.
**E06**	Female	83/Sant Martí	6 (Moderate)	7 (Vulnerable)	Replaced family dinners with sweet comfort foods and continuous TV background.	**Concordant:** Emotional loneliness driving single-plate carbohydrate dependency.
**E07**	Female	71/Sant Andreu	6 (Moderate)	8 (Vulnerable)	Family supply of olive oil and nuts, but personal intake collapses to sandwiches.	**Discordant:** External nutritional support is structurally present but behaviorally unexploited.
**E08**	Male	79/Sants	5 (Low)	−1 (Risk)	Severe cooking aversion; exclusive use of microwave for industrial frozen portions.	**Concordant:** Male skill deficit combined with low motivation leads to a total collapse of the kitchen.
**E09**	Male	87/Les Corts	8 (Moderate)	9 (Promotor)	High informal support; daughter cleans and preps fresh vegetables weekly.	**Discordant:** Adequate intake sustained through surrogate care, masking low intrinsic motivation.
**E10**	Male	81/Nou Barris	6 (Moderate)	3 (Vulnerable)	Severe geographical and pension limitations; reliance on cheap processed fish.	**Concordant:** Socioeconomic constraints restrict the purchase of fresh Mediterranean proteins.
**E11**	Female	80/Sarrià	5 (Low)	5 (Vulnerable)	Intermittent fasting and exclusive batch-preparation of Thermomix purées.	**Concordant:** Drastic menu reduction down to a single, hyper-simplified food format.
**E12**	Male	75/Nou Barris	5 (Low)	−2 (Risk)	Severe post-widowhood depressive apathy; absolute neglect of kitchen spaces.	**Concordant:** Deep grief-induced abandonment of both dietary quality and physical order.

## 4 Discussion

### 4.1 Principal findings

The findings of this mixed-methods study demonstrate that the transition to living alone among individuals over 65 years of age in the city of Barcelona transforms food acquisition routines, culinary preparation, and dietary intake patterns. These results reinforce existing evidence indicating that social isolation and loneliness are determinant factors negatively associated with dietary behavior and quality in old age (30). The evidence obtained underscores the need to understand eating alone not merely as a nutritional risk factor, but as a complex phenomenon that threatens adherence to the Mediterranean Diet pattern (31–33).

Previous literature on home food inventories suggests that high physical availability of healthy foods in the home usually correlates with a higher consumption of these foods (17). However, the introduction of involuntary loneliness as a variable alters this premise. The data triangulation in this study evidences the existence of a discordance, wherein having promoting infrastructures and healthy foods in the refrigerator or pantry does not guarantee their optimal consumption. Qualitative narratives explain this phenomenon through culinary “apathy” and the systematic avoidance of cooking daily for a single person. This pushes older adults toward extreme pragmatic simplification, leading to an overreliance on the microwave, kitchen robots (for purées or easy cold soups, like gazpacho), or the purchase of convenient ready-to-eat meals. The coexistence of home-prepared food containers and microwave usage represents a double-edged duality. For participants experiencing active and resilient aging, batch cooking allows them to preserve the Mediterranean pattern (legumes, vegetables) while optimizing time and physical effort. For them, the microwave serves as an easy ally (34) in home safety that minimizes the risk of burns or gas leaks.

As Freire et al. (10) points out, loneliness affects eating habits in different ways, including a reduced interest in food acquisition, preparation, and consumption, as well as decreased satisfaction with mealtimes, ultimately leading to a diminished perception of personal quality of life. These results are consistent with previous research indicating that cooking for oneself often leads to the adoption of monotonous, functional survival diets (14). Commensality acts as a strong protective factor (4, 32). The maintenance of high-quality diets requires social incentives (35). Recent reviews confirm that factors such as emotional support and social connections are determinants that facilitate a higher adherence to the Mediterranean Diet in adults (36).

The act of eating transcends caloric needs to establish itself as a fundamental social structurer (37). The Mediterranean Diet is characterized not only by “what” is eaten, but by “how” and “with who” it is eaten (33), with conviviality (35) and commensality (the pleasure of sharing the table with significant others) being a fundamental pillar of this cultural pattern (38). Our results show that living and eating alone blurs the symbolic value of food. This phenomenon interacts directly with the concept of “culinary solitude,” where the absence of an “other” with whom to share generates feelings of isolation (39, 40) and apathy, that disincentivize the preparation of laborious dishes, or even eating in some occasions.

In our sample, the category of “social foods” (e.g., paella, cannelloni, seafood) and social beverages like wine has strongly emerged, as their solitary consumption is abandoned because they lack context, or they generate shame or sadness. As recent ethnographic studies on loneliness in older people suggest (10), food (including drinks) requires a context of commensality to legitimize its hedonic enjoyment. Likewise, faced with the emotional weight of silence in the home, older adults deploy coping strategies such as the use of sound accompaniment, routinely turning on the television or radio during meals (30). However, screen exposure during meals has been linked to lower adherence to the Mediterranean diet and higher consumption of ultra-processed foods (41).

The experience of dietary solitude is not homogeneous and presents gender differences, in line with other studies (10, 42, 43). For women, the extreme simplification of cooking represents a complex negotiation between apathy and liberation. Having spent decades tied to the domestic obligation of feeding a family, living alone allows them to escape from a history of family-oriented food service. The transition to a single-plate format or quick purées also represents a reclamation of autonomy. However, this liberation carries a nutritional cost: when the caregiving role is deconstructed, the motivation to sustain a diverse diet diminishes.

Conversely, male participants display a pattern of structural vulnerability rooted in a generation-specific lack of domestic skills. Widowed or single men frequently face a steep learning curve due to a lifetime of reliance on female partners for food management. This dynamic explains why the lowest scores for Mediterranean diet adherence and risk home food environments occurred exclusively among men (E08, E12). While women maintain domestic infrastructure through deeply ingrained cleaning and sorting habits despite experiencing equal loneliness, men show a higher risk of total behavioral abandonment, shifting rapidly to canned food or automated microwave-dependent ultra-processed diets.

It must be also noted that, among profiles exhibiting symptoms of depressive apathy (such as E08 and E12), the microwave acts as a facilitator that perpetuates the replacement of traditional cooking with easy-open, ultra-processed meals. The disappearance of traditional cooking utensils and the dominance of single-use plastics on the countertop reflect a process of culinary dispossession, wherein the home loses its capacity to generate health.

Complementarily, as evidenced by Edfors and Westergren (44), habits, preferences, and cultural norms formed in early youth have a lasting impact on how older adults view food. However, economics or declining mobility force a radical restructuring of food acquisition. As studies on active aging in metropolitan environments point out (45), distances to food stores become a critical barrier for older adults with reduced mobility, since many prefer daily in-person shopping as a socialization tool, firmly rejecting digital environments or online shopping. Furthermore, material vulnerability aligns with findings on how socioeconomic crises and low purchasing power erode dietary quality in the Mediterranean environment, radically modifying lifestyles (46). Although the Mediterranean pattern is considered of high value, some retirees define their daily economy as “rigid,” restricting access to traditional fresh protein sources (quality fish and meat) and opting for more affordable ultra-processed alternatives.

The combination of social isolation, menu destructuring, and low consumption of fresh produce threatens physiological sustainability in old age. Literature shows that the loss of the Mediterranean pattern and malnutrition secondary to loneliness are direct precursors of sarcopenia, involuntary weight loss, and frailty in older adults (47). Conversely, high adherence to diets rich in olive oil, nuts, vegetables, and fish, such as the Mediterranean Diet, has been shown to have strong neuroprotective and antioxidant properties, mitigating age-associated chronic inflammation and reducing the risk of cognitive decline and cardiovascular disorders (48). However, as observed in population studies, older adults living alone often establish a “normative silence,” minimizing the true risks that loneliness and lack of resources pose to their nutritional status, which hinders early detection by the healthcare system (49). On the other hand, it should be noted that this loss of adherence is also related to the fact that people stop consuming traditional dishes that are part of their family and taste memories (comfort foods). This also implies a weaker connection with what they eat and can exacerbate psychological problems related to depression and a lack of will to live (50).

### 4.2 Strengths and limitations

This study may show limitations inherent to its qualitative cross-sectional design. Regarding the size and composition of the sample, although discursive saturation was achieved, it is possible that the information may not capture sufficient diversity of perspectives. A certain degree of bias may also be present regarding a sample limited to a specific urban geographical environment (Barcelona), which restricts the generalizability of the findings to an urban context. As such, the dynamics observed in this metropolitan setting are expected to differ significantly from those in rural contexts or smaller towns. In rural Mediterranean communities, informal social safety nets, tighter neighborhood cohesion, and traditional food-sharing practices—such as access to family agriculture or home gardens—may serve as protective buffers against the dietary apathy induced by solitary living (36, 51). Furthermore, empirical evidence in Spain confirms notable rural-urban differences in older adults' social support networks and life satisfaction (52). Therefore, comparing these urban findings with rural cohorts remains a crucial next step to fully understand how different spatial and community environments modulate the impact of involuntary loneliness on dietary sustainability, which is precisely the aim of our ongoing broader research project.

Additionally, the HFE-MED-PHOTO index is an *ad hoc* adapted instrument for the visual analysis of the home in older adults within the Mediterranean context. Although its content validity is governed by an evaluation based on theoretical criteria (53)—emulating the methodological principles of validated home inventories in the literature (17)—it would require psychometric validation for future studies.

Whilst a process of triangulation was applied throughout the analytical phase, the potential impact of the researchers' personal conceptual frameworks cannot be fully discounted. Loneliness is occasionally linked to the stigma of abandonment, and this may trigger tendencies toward social desirability and the narrative reshaping of accounts, both of which require careful consideration. Lastly, because this study investigates the effect of loneliness on dietary behaviors using qualitative interviews with individuals situated in particular life circumstances (such as varying durations of isolation, differing degrees of acceptance, and distinct underlying causes), these elements may serve as confounding variables. Consequently, further studies reproducing this research across alternative geographical regions (in Spain, but not only) and different contexts (urban, rural) remain essential.

Another potential bias may be related to gender and sexual orientation in the selection of interviewees, especially regarding men. Some studies indicate that cisgender heterosexual men demonstrate lower culinary skills than, for example, cisgender gay men (54). In this study, all the men interviewed identified as heterosexual. Expanding the sample to include more diverse individuals could yield interesting results and conclusions in this regard.

Nevertheless, and at the same time, a key strength of this study lies in the triangulation of its mixed-methods design. By combining in-depth qualitative narratives with validated dietary adherence indices and the objective visual analysis of the physical home food environment, recall and social desirability biases—which are common in conventional dietary questionnaires—are significantly reduced. Furthermore, the adoption of a phenomenological perspective gives a voice to older adults, allowing for the firsthand capture of the complex emotional, gendered, and sociocultural dimensions of loneliness that often remain invisibilized in purely epidemiological research (10).

These issues are critical for the design of social and health policies. Interventions aimed at improving the nutritional status of older adults living alone will prove ineffective if they are limited solely to nutritional education. Promoting the Mediterranean Diet in this population group inevitably requires addressing its social and cultural dimension.

## 5 Conclusions

The findings of this study demonstrate that transitioning to living alone in old age fundamentally alters food acquisition, culinary routines, and dietary quality, primarily by threatening adherence to traditional foods, frequently based in a defined Mediterranean diet pattern. Rather than operating merely as a physical risk factor, involuntary loneliness introduces a critical discordance where the physical presence of healthy food in home inventories fails to guarantee its consumption. If commensality is the shared social experience that historically legitimizes hedonic enjoyment and culturally gives structure to mealtimes, the absence of commensality leads to culinary apathy, the abandonment of traditional ‘social foods', and a reliance on monotonous, pragmatic survival diets. Consequently, older adults increasingly resort to extreme culinary simplification, over-utilizing convenience options, ultra-processed items, and voice sound eclectic accompaniment (such as television or radio) to offset the emotional weight of solitary dining.

This experience of dietary solitude is deeply nuanced by gender and socioeconomic constraints. For women, simplifying culinary tasks often reflects a complex negotiation between domestic liberation and a diminished motivation for self-care. On the other hand, men exhibit structural vulnerability stemming from a generational deficit in domestic skills, leaving them at a higher risk of total behavioral abandonment and reliance on ultra-processed diets. Furthermore, material limitations, reduced mobility, and rigid retirement budgets compound these challenges by restricting physical access to fresh produce and forcing a reliance on cheaper alternatives. Together, these socio-emotional and structural barriers accelerate menu destructuring, which directly threatens physiological and psychological sustainability, increasing susceptibility to sarcopenia, frailty, and cognitive decline, while a “normative silence” often conceals these emerging health risks from clinical detection.

Ultimately, these results indicate that public health and social interventions cannot rely solely on nutritional education. Protecting the dietary health of older adults living alone requires comprehensive community strategies that address the socio-cultural dimensions of eating, fostering spaces of shared commensality and rebuilding social connections to ensure that nutritional support addresses both physical nourishment, cultural cogency and social wellbeing (55).

It is essential to promote interdisciplinary research and action, and also develop community health strategies that mitigate loneliness, reactivate motivation for self-care, and foster spaces of shared commensality, recognizing that for interventions aimed at improving the nutritional health of older adults living alone require the integration of social, environmental, and community support factors to maximize their effectiveness.

## Data Availability

The datasets presented in this article are not readily available because the datasets generated during the current study are available from the corresponding author on reasonable request, subject to the restrictions of the informed consent provided by the participants. Requests to access the datasets should be directed to FM, fxmedina@uoc.edu.
