# Supplementary material for: Unwanted loneliness, food practices, and Mediterranean diet adherence among older adults in Barcelona city (Spain)
Source: Front Nutr. 2026 Oct 8;13:1947655. doi: 10.3389/fnut.2026.1947655 (PMC13687317; doi:10.3389/fnut.2026.1947655)
Supplement: Supplementary file 1 [file Table_1.docx]

***Supplementary Material***

# 1 Supplementary Figures and Tables

## 1.1 Supplementary Table

Coding matrix and scoring criteria of the HFE-MED-PHOTO tool (Home Food Environment photo-audit for Mediterranean older adults).

This document details the visual instrument used by independent raters for the objective audit of photographs of refrigerators, pantries, and countertops of participants aged 65 and over.

**Table S1. Evaluation Protocol and Item Scoring Criteria**

| **Code** | **Block / Area** | **Variable to Evaluate** | **Scoring Criteria** | **Coding Notes for the Evaluator** |
| --- | --- | --- | --- | --- |
| **A1** | **Refrigerator** | Fresh market vegetables | **[+2]** If ≥ 3 different types.  **[+1]** If 1–2 types.  **[0]** Total absence of fresh vegetables. | Prioritize evaluating the lower crisper drawer and central shelves. |
| **A2** | **Refrigerator** | Refrigerated fresh fruit | **[+2]** If ≥ 2 different types.  **[+1]** If only 1 type.  **[0]** Total absence. | Prioritize evaluating the lower crisper drawer and central shelves. |
| **A3** | **Refrigerator** | Fermented dairy products | **[+1]** Unsweetened plain yogurt or traditional cheeses (fresh, semi-cured).  **[-1]** Only sweetened dairy desserts (flans, custards).  **[0]** No dairy. | Key indicator in older populations for monitoring calcium intake. |
| **A4** | **Refrigerator** | Healthy Protein | **[+1]** Presence of eggs, fish, or white meat (chicken, turkey, rabbit).  **[0]** Absence of unprocessed fresh protein. | Evaluate the presence of raw ingredients for cooking. |
| **A5** | **Refrigerator/ Kitchen** | Processed protein and Ready-to-eat meals | **[-1]** Presence of low-cost cured meats, processed meats, or industrial microwave-ready meals.  **[0]** Absence of these products. | Measures the penetration of convenience food replacing traditional cooking. |
| **A6** | **Refrigerator/ Kitchen** | Sugar-sweetened beverages or Alcohol | **[-1]** Presence of soft drinks, packaged juices, beer, wine, or spirits.  **[0]** Absence of these beverages. | Identified as a risk factor in isolated older adults. |
| **B1** | **Countertop** | The Fruit Bowl (*Nudging*) | **[+1]** Visible fruit bowl with accessible fruit.  **[0]** No fruit bowl or empty. | Measures physical accessibility and visual incentives for direct fruit consumption. |
| **B2** | **Countertop** | Dressing and cooking fat | **[+1]** Presence of Olive Oil (any variety).  **[0]** Absence of olive oil. | Rewards exclusively the traditional Mediterranean fat pillar adjacent to the stove area. |
| **B3** | **Countertop** | "Trigger" Ultra-processed foods | **[-2]** Industrial pastries, cookies, or potato chips visibly displayed.  **[0]** Area clear of visible unhealthy snacks. | Measures unfavorable decision architecture and factors associated with disordered appetite. |
| **C1** | **Pantry** | Legumes (Med Core) | **[+1]** Presence of dried or plain canned cooked legumes.  **[0]** Absence of legumes. | Verify the presence of this core source of plant-based protein staple in the pantry. |
| **C2** | **Pantry** | Traditional canned fish | **[+1]** Presence of canned tuna, sardines, mackerel, or cockles.  **[0]** Absence. | Traditional, healthy, long-shelf-life protein resource for older adults. |
| **C3** | **Pantry** | Cereals (Rice, bread, pasta) | **[+2]** Present in whole grain version.  **[+1]** Present only in refined/white version.  **[0]** Absence of basic cereals. | Qualitatively evaluates the presence of whole-grain dietary fiber. |
| **C4** | **Pantry/ Countertop** | Nuts | **[+1]** Presence of raw or roasted nuts.  **[0]** Total absence. | Healthy, cardioprotective snack typical of the Mediterranean diet. |
| **D1** | **Refrigerator** | Leftover management | **[+1]** Presence of tupperware containing home-cooked meal preparations.  **[0]** Absence of stored home-cooked food. | Direct indicator of maintaining active home cooking. |
| **D2** | **Refrigerator/ Countertop** | Packaging sustainability | **[+1]** Reused glass jars or durable food containers.  **[-1]** Predominance of single-use plastics or disposable supermarket packaging. | Measures traditional sustainable food preservation versus convenience culture. |
| **D3** | **Kitchen** | Microwave Usage | **[+1]** Microwave coexists with tupperware containing home-cooked food.  **[-1]** Microwave coexists exclusively with supermarket pre-cooked meals.  **[0]** No microwave available or its usage is neutral. | Evaluates whether technology acts as a support for autonomy or as a dietary barrier. |

The HFE-MED-PHOTO score was developed as an ad-hoc, exploratory visual audit tool. Content validity was established by mapping its items directly against the validated 14-item MEDAS screener (1) and validated Home Food Inventories (2). The a priori scoring system followed established methodological frameworks for diet quality indices (3).

- Risk Environment (≤ 1point): Represents a critical scenario where penalties for ultra-processed foods or the systematic absence of fresh produce neutralize any baseline protective factors in the environment. Mathematically, this score precludes the coexistence of essential fresh foods, identifying hostile or depleted kitchen environments.
- Promoting Environment (≥ 9 points): This threshold was designed under a minimum-requirements model for the Mediterranean dietary pattern. For a participant to achieve a score of ≥ 9 or higher on a scale that penalizes processed items, the index algorithm mathematically requires the simultaneous fulfillment of multiple health pillars: a variety of fresh vegetables, fresh fruit, olive oil, legumes, canned fish, nuts, and favorable logistical organization such as home-cooked food containers, ensuring that the physical household infrastructure is truly health-promoting.
- Vulnerable Environment (2 to 8 points): Defines a transition zone or culinary resistance. The household maintains basic non-perishable Mediterranean Diet resources (grains, oils, canned legumes) but lacks a steady inflow of daily fresh produce or is shadowed by the moderate intrusion of convenience items.

### References

1. [Schröder H, Fitó M, Estruch R, Martínez-González MA, Corella D, Salas-Salvadó J, et al. A Short Screener Is Valid for Assessing Mediterranean Diet Adherence among Older Spanish Men and Women. J Nutr. 2011 Jun;141(6):1140–5. doi:10.3945/jn.110.135566](https://www.zotero.org/google-docs/?LFlM6p)
2. [Fulkerson JA, Nelson MC, Lytle L, Moe S, Heitzler C, Pasch KE. The validation of a home food inventory. Int J Behav Nutr Phys Act. 2008;5(1):55. doi:10.1186/1479-5868-5-55](https://www.zotero.org/google-docs/?LFlM6p)
3. [Burggraf C, Teuber R, Brosig S, Meier T. Review of a priori dietary quality indices in relation to their construction criteria. Nutr Rev. 2018 Oct 1;76(10):747–64. doi:10.1093/nutrit/nuy027](https://www.zotero.org/google-docs/?LFlM6p)
